# Production and Perception of Tone 3 Focus in Mandarin Chinese

**DOI:** 10.3389/fpsyg.2016.01058

**Published:** 2016-07-26

**Authors:** Yong-Cheol Lee, Ting Wang, Mark Liberman

**Affiliations:** ^1^Department of English Language and Literature, Cheongju UniversityCheongju, South Korea; ^2^School of Foreign Languages, Tongji UniversityShanghai, China; ^3^Department of Linguistics, University of PennsylvaniaPhiladelphia, PA, USA

**Keywords:** tone 3, corrective focus, prosody, pre-low raising, post-low bouncing

## Abstract

This study uses production and perception experiments to explore tone 3 focus in Mandarin Chinese. Overall, contrastive focus in Mandarin is clearly marked with increased duration, intensity, and pitch range: in the experiments, listeners identified focused syllables correctly more than 90% of the time. However, a tone 3 syllable offers a smaller capacity for pitch range expansion under focus, and also yields less intensity increase; in addition, local dissimilation increases the duration, intensity, and pitch range of adjacent syllables within the same phrase as a focused tone 3 syllable. As a result, tone 3 focus was less well identified by listeners (77.1%). We suggest that the relatively poor identification of tone 3 focus is due to the smaller capacity for pitch range expansion, the confusion from within-phrase local dissimilatory effects, and the relatively weak intensity of tone 3. This study demonstrates that even within a language where purely prosodic marking of focus is clear, the location of prosodic focus can be difficult to identify in certain circumstances. Our results underline the conclusion, established in other work, that prosodic marking of focus is not universal, but is expressed through the prosodic system of each language.

## Introduction

Focus represents the most important message in a sentence (Halliday, 1967). Due to its important communicative function, focus is encoded by prosodic means in many languages by inducing a long duration, high intensity, high mean pitch, and a large pitch range. In Mandarin, focus carries such prosodic correlates, but the prosodic realization of focus actually differs by tone (Shih, 1988; Xu, 1999). For example, focus raises the pitch of a high tone (tone 1) but lowers the pitch of a low tone (tone 3; Xu, 1999; Cao, 2002, 2012), indicating that unlike the universal phonetic symbolism of focus that raises pitch, tone 3 focus is characterized by lowering a pitch target. An important issue here is whether such a downward pitch movement is sufficient in cueing focus. If it is not sufficient, which parameters then play key roles in tone 3 focus?

Before setting up the research goals, we first survey relevant work to this study by providing a brief overview of lexical tones in Mandarin Chinese, which are important in understanding the details of the study. We then review the literature on the production and perception of prosodic focus in Mandarin Chinese. Finally, the research goals of this study will be presented based on the review of relevant literature.

### A brief overview of four lexical tones in mandarin chinese

Mandarin consists of four lexical tones: a high level tone (tone 1), a rising tone (tone 2), a low/dipping tone (tone 3), and a falling tone (tone 4). These tones are used to contrast homophonous morphemes, as illustrated in (1). Tones 1–4 are conventionally labeled as [55], [35], [214], and [51] depending on the pitch level, where [1] represents the lowest pitch level, and [5] the highest pitch level (Chao, 1968).

(1) a. /ma/ with tone 1 ⇒ “mother”
b. /ma/ with tone 2 ⇒ “hemp”
c. /ma/ with tone 3 ⇒ “horse”
d. /ma/ with tone 4 ⇒ “to scold” (Xu, 1997, p. 64)

Furthermore, Mandarin Chinese includes two basic kinds of pitch targets associated with tones: static and dynamic (Xu and Wang, 2001). There are two static (low and high) and two dynamic (rising and falling) pitch targets: tone 1 has a high pitch target; tone 2 has a rising pitch target from low to high; tone 3 has a low pitch target; and tone 4 has a falling pitch target from high to low. Based on their pitch targets, tones 1–4 can be broadly classified into two groups, one in which tones 1, 2, and 4 have a high pitch point, and the other in which tone 3 lacks a high pitch point.

### Production and perception of focus in mandarin chinese

It has been observed that focus involves different kinds of prosodic adjustments that differ depending on whether they occur in focus, post-focus, or pre-focus position. In the focus position, focus increases duration, intensity, and pitch range (Xu, 1999; Wang et al., 2002; Yuan, 2004; Liu and Xu, 2005; Chen and Gussenhoven, 2008). In post-focus positions, duration, intensity, and pitch range are considerably compressed (Xu, 1999; Yuan, 2004; Liu and Xu, 2005), which is known as post-focus compression (Chen et al., 2009; Xu et al., 2012). Yet these parameters show no significant changes in pre-focus positions (Liu and Xu, 2005; Xu, 1999; Yuan, 2004).

As previously stated, tone 3 focus is expressed in a unique fashion by lowering a pitch target. Several studies have attempted to ascertain the prosodic characteristics of tone 3 focus, but no clear picture has been obtained of its exact nature. Shih (1988) argues that it is unclear whether the low pitch target is actually lowered under focus. On the other hand, other studies claim that focus does lower the low pitch target of a tone 3 syllable (Chao, 1968; Xu, 1999; Chen and Gussenhoven, 2008; Cao, 2012). A different view is that a long duration plays an important role in cueing tone 3 focus (Wang, 2002). Regarding pre- and post-focus effects, tone 3 focus involves (unique) local dissimilatory effects: pitch becomes raised immediately before focus, known as pre-low raising (Xu and Wang, 2001; Liu and Xu, 2007); and pitch bounces back immediately after focus, known as post-low bouncing (Liu and Xu, 2007; Prom-on et al., 2012).

Moving on to perception, it has been attested that focus identification does not differ by tone but does differ by position (Yuan, 2004; Liu, 2009). Yuan (2004) found that the ordering of identification rates from highest to lowest was sentence-medial (92.9%) > sentence-initial (87.2%) > sentence-final (75.5%), where the symbol “>” indicates a significant difference. In Liu (2009), the results revealed a similar result: sentence-medial (97.2%), sentence-initial (95.3%) > sentence-final (82.6%). Regarding the perceptual cues for focus, shifting pitch contours and raising a high pitch target are important cues, although the latter plays a more important role (Wang et al., 2002). Moreover, a large body of evidence has demonstrated that post-focus compression serves as a highly effective perceptual cue for focus (Xu et al., 2004, 2012; Liu and Xu, 2005; Chen et al., 2009).

In contrast to other focused tones, tone 3 focus draws relatively little attention in studies of perception—to our knowledge, there have been only two studies that attempted to examine the perception of tone 3 focus (Yuan, 2004; Cao and Zhang, 2008). In the case of Cao and Zhang's (2008) experiment, stimuli were synthesized in three separate positions (sentence-initial, sentence-medial, sentence-final), where duration, creakiness, and pitch range were incremented during each step in order to approximate the natural prosody of tone 3 focus. The findings indicated that creakiness is important in sentence-initial position, whereas lengthening is important in sentence-final position. Finally, they concluded that the most important cue for tone 3 focus is a mid-sized pitch drop (6 semitones), although creakiness and lengthening improve identification in some positions. Using natural stimuli, Yuan (2004) reported that the ordering of identification rates of tone 3 focus is congruent with the identification of other focused tones, i.e., sentence-medial > sentence-initial > sentence-final.

### The current study

From a literature review, we observe several limitations in the stimuli of previous work. First, in many studies, a tone 3 syllable was excluded from the stimuli (e.g., Xu, 1999; Wang and Xu, 2011; Kabagema-Bilan et al., 2011), presumably due to tone sandhi—tone 3 becomes tone 2 when followed by another tone 3. Second, although some studies indeed included a tone 3 syllable in the stimuli (e.g., Liu, 2009; Greif, 2010), it seems that the full scale of tone 3 focus was (largely) masked by the structural limitations inherent in the stimuli. For example, in the stimuli of Greif (2010), the name *Ma3 Long2* was designed to be contrastively focused, as shown in (2). In this case, although the entire sequence was in the domain of semantic focus, the tone 2 syllable seemed to carry main prominence (via focus).

(2) Q: Has Tom got two or three watermelons?
A: Bu4, [Ma3 Long2]_F_ you3 xi1 gua1.
“No, Marlon has the watermelons.”

In Liu (2009), two consecutive tone 3 syllables (e.g., *Li3 Min3*) were designed to receive focus, but the sequence changed to *Li2 Min3* due to tone sandhi. As a result, similar to the case of Greif (2010), the tone 2 syllable seemed to carry main prominence. The phenomena described here are similar to the case where focus is encoded by a primary stressed syllable in a multisyllabic word in English (Ladd, 1996; Cohan, 2000), although the whole word is in the focus domain. Therefore, we need an experiment design where tone sandhi is avoided, and at the same time a tone 3 syllable is marked by prosodic prominence. Third, although Liu and Xu (2007) discovered the local dissimilatory effects of tone 3 focus, the distribution of tone 3 focus was fairly restricted in their stimuli—only the second and third words in a sentence alternately contained tone 3 focus, which leads us to explore the local dissimilatory effects in a full scale. Finally and most importantly, local dissimilatory effects have not yet been studied in perception.

Our understanding of tone 3 focus is far from complete due to the limited investigation of it in existing studies, whether relating to production or perception. There are some important issues that need to be considered. First, it is unclear whether a downward pitch movement with tone 3 focus is a perceptually sufficient cue for listeners. Second, we do not know yet whether pre-low raising and/or post-low bouncing are independent of focus position: do they only appear within the same prosodic phrasing or are they still visible across the phrase boundary? Given that pitch normally resets after the phrase boundary, it is likely that local dissimilatory effects appear within the same phrase. The third issue concerns the role of local dissimilatory effects in perception: do they help listeners perceive tone 3 focus or hinder listeners' perception? With these issues in mind, this study attempts to achieve two research goals: (a) to determine the nature of tone 3 focus and its local dissimilatory effects; and (b) to examine whether listeners can successfully identify tone 3 focus or whether local dissimilatory effects hinder the recognition of tone 3 focus.

Depending on the situation in which focus is used, it can be divided and labeled into several types, three of which are discourse-new focus, contrastive focus, and corrective focus. We designed stimuli that produced corrective focus within a paradigm of 10-digit phone-number strings and conducted production and perception experiments to achieve the goals of the current study.

## Production

### Stimuli

A Python script created randomized 100 10-digit strings, designed such that each digit occurs equally often in each position, and each pair of digits occurs equally often across each pair of positions. Please note that tone sandhi was avoided for target digits in the stimuli. The 100 10-digit strings were produced in two focus conditions: broad focus and corrective focus^1^. Target strings were produced in isolation for broad focus, as in (3a). The same sequences were embedded in a Q&A dialogue for corrective focus, as in (3b), where a questioner confirms whether the phone-number is correct, and a speaker responds to the question by correcting the wrong digit.

(3) a. Broad-focus condition
  李梅的电话号码是 787-412-4699。 “Li Mei's number is 787-412-4699.”
b. Corrective-focus condition
Q: 李梅的电话号码是 887-412-4699 。是吗？ “Li Mei's number is 887-412-4699. Right?”
A: 不是。李梅的电话号码是 787-412-4699。 “No, Li Mei's number is 787-412-4699.”

Table 1 introduces the numerical digits (0–9) of Mandarin. Among the 10 digits, there are four tone 1 digits (1, 3, 7, 8); one tone 2 digit (0); two tone 3 digits (5, 9); and three tone 4 digits (2, 4, 6).

**Table 1:** **The numerical digits of Mandarin, with Pinyin romanization, and the lexical tone of each digit**.

**Digit**	**Pinyin romanization**	**Lexical tone**
0	Ling	Tone 2
1	Yi	Tone 1
2	Er	Tone 4
3	San	Tone 1
4	Si	Tone 4
5	Wu	Tone 3
6	Liu	Tone 4
7	Qi	Tone 1
8	Ba	Tone 1
9	Jiu	Tone 3

### Subjects

We recruited two male and three female graduate students (mean age: 25.2, SD: 1.1) at the University of Pennsylvania who had been in the US for less than a year at the time of recording. All the speakers were native Mandarin speakers with no speech or hearing impairments. Speakers signed a consent form and received 10 dollars as compensation.

### Recording procedure

Recordings were conducted in a sound-attenuated booth at the University of Pennsylvania and were saved directly to a laptop using a Plantronics headset microphone at 44.1 kHz with 16 bits per sample. During the recordings, stimuli were presented to speakers through PowerPoint slides.

Speakers were seated in front of a laptop monitor, wearing a headset microphone. In the beginning of the recording session, we presented speakers with three practice trials for each of the two focus conditions to familiarize them with the recording procedure. During the actual recording sessions, speakers first read 100 10-digit strings in isolation for broad focus, and (after a short break) they listened to pre-recorded questions through headphones and read the target strings as answers for corrective focus. The recording time was around 15 min for broad focus and around 25 min for corrective focus.

In total, we collected 1000 10-digit strings (100 strings × 5 speakers × 2 focus conditions). Then, by dividing the strings by each tone, we collected 400 strings for tone 1, 100 strings for tone 2, 200 strings for tone 3, and 300 strings for tone 4. This particular breakdown of proportions is based on the fact that, as shown in Table 1, there are four tone 1 digits, one tone 2 digit, two tone 3 digits, and three tone 4 digits in Mandarin. Therefore, we have such proportions for each tone.

### A sketch of pitch contours

Before analyzing the data, we illustrate some of the pitch contours that enable us to capture the overall picture of tone 3 focus, and then move on to the pitch contours that portray prosodic characteristics of each focused tone. In this study, the pitch contours were sampled at 10 equidistant points of each labeled digit using ProsodyPro (Xu, 2013).

**Figure 1:**
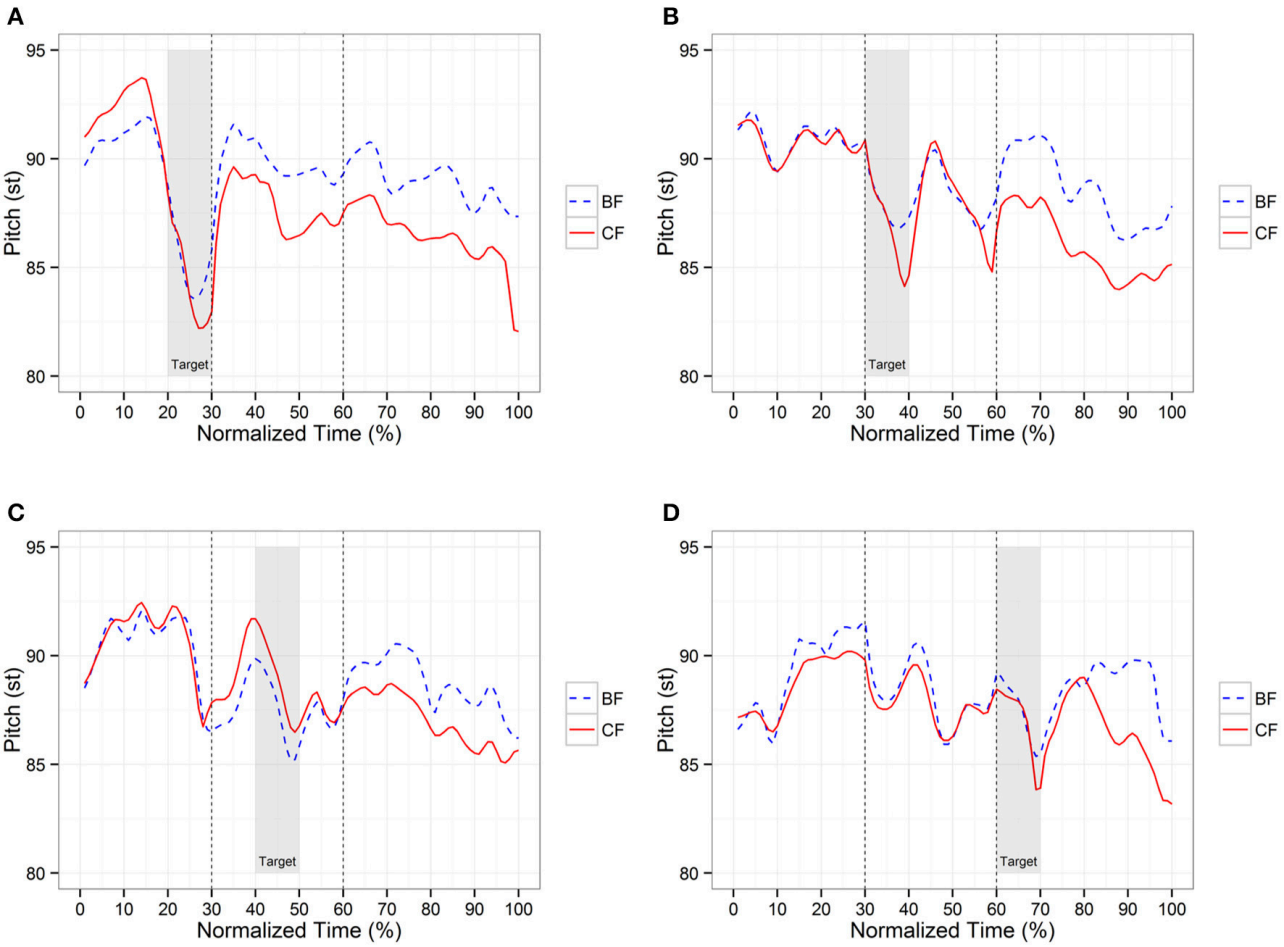
**Sample pitch contours for tone 3 digits in two focus conditions**. BF and CF are abbreviations for broad focus and corrective focus, respectively. The shaded area in gray represents the focus position and vertical lines refer to phrase boundaries.

Figure 1 displays time-normalized pitch contours averaged by five speakers for tone 3 digits, where the shaded area in gray represents the focus position and vertical lines refer to phrase boundaries^2^. From Figure 1, we can observe that the corrective-focus condition shows a more expanded pitch range than its broad-focus counterpart in the focus position. At the same time, we can observe noticeable differences in the pre- and post-focus positions. In Figures 1A,C, the corrective-focus condition shows a higher level of pitch in the pre-focus position (i.e., pre-low raising), whereas Figures 1B,D show no such thing in the same position. Regarding the post-focus effect, only Figure 1A shows clearly compressed pitch contours (i.e., post-focus compression) right after focus. In Figures 1B–D, post-focus compression is not visible right after focus; rather, the pitch bounces up after a very low pitch (i.e., post-low bouncing), indicating that post-focus compression is absent where post-low bouncing is present. It seems that local dissimilatory effects like pre-low raising and post-low bouncing are position-dependent—they are present within the same phrase but absent across the phrase boundary.

**Figure 2:**
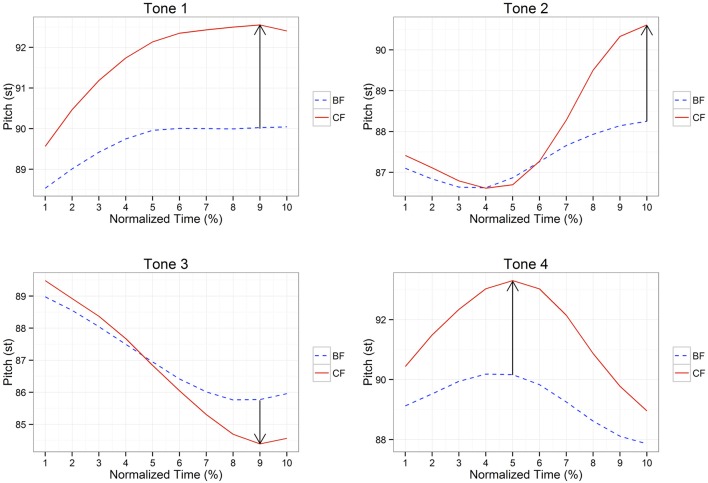
**Pitch trajectories of tones 1–4 in two focus conditions**. BF and CF are abbreviations for broad focus and corrective focus, respectively. The upward arrow represents that focus is expressed by raising its pitch target, whereas the downward arrow indicates that focus is expressed by lowering its pitch target.

Figure 2 exhibits the pitch trajectories of tones 1–4 in two focus conditions aggregated by 10 string positions. As demonstrated in Figure 2, corrective focus is marked similarly by greater pitch range expansion for all tone types, which is captured by the rise/fall size, subtracting the high/low pitch point of broad focus from that of corrective focus. However, there appear two noticeable differences between tone 3 and other tones. One is that tone 3 focus is characterized by lowering a low pitch point, but other focused tones are realized by raising a high pitch point. The other is that unlike other focused tones, tone 3 focus seems to have a smaller capacity for pitch range expansion.

**Figure 3:**
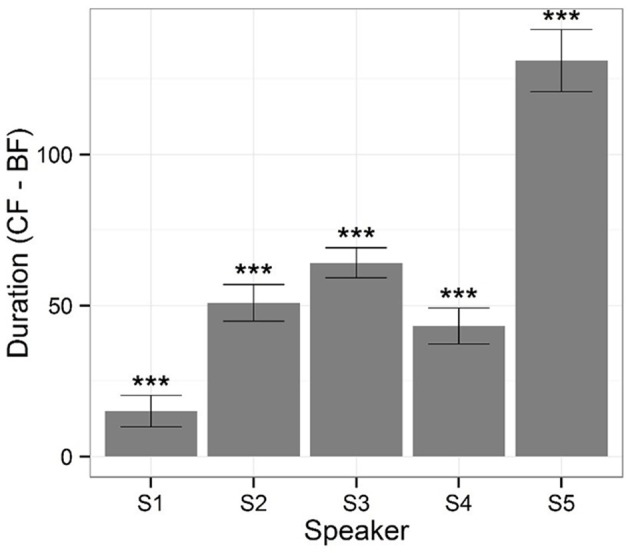
Bars represent differences in duration calculated by corrective focus minus broad focus by each speaker and error bars indicate 95% confidence intervals (^***^p < 0.001).

### Acoustic measurements

Based on the visual observations of Figures 1, 2, acoustic measurements were conducted from three different areas: focus, pre-focus, and post-focus. In the focus position, we measured duration in milliseconds (ms) and mean intensity in decibels (dB) of each labeled digit to directly compare the two focus conditions. Furthermore, in order to estimate the size of pitch range expansion between broad and corrective focus, we measured maximum pitch in semitones (st) for tones 1, 2, and 4 and minimum pitch in semitones (st) for tone 3, which are assumed to best reflect the underlying pitch target of each tone. Please note that pitch range indicates the difference in values between maximum and minimum pitch, and the semitone scale was obtained by applying the following formula (Xu and Wang, 2009; Chen, 2012):

$$\begin{array}{l} Semitone=12log_{2}x \end{array}$$

where *x* indicates a raw f0 value in hertz (Hz), and 1 Hz is used as a reference value. In this study, we label the maximum and minimum pitch as “target pitch” for simplicity's sake. In pre- and post-focus positions, we measured duration (ms), mean intensity (dB), and mean pitch (st) from the positions immediately preceding and following focus to analyze the local dissimilatory effects of tone 3 focus. After carefully checking and manually correcting vocal pulse markings, we automatically obtained these measurements by implementing ProsodyPro (Xu, 2013), based on hand-labeled digit boundaries.

### Analyses and results

The basic analysis strategy was to make a direct comparison between the broad-focus and corrective-focus conditions, which are separated by focus position: focus, pre-focus, and post-focus. In the focus position, we examined the tone 3 digits by the aggregate measures of duration, mean intensity, and target pitch, and also included the other tones for reference data. In pre-focus positions, given that pre-low raising seems to occur only within the phrase, we divided the string position into two parts: final vs. non-final positions. In the 10-digit string (NNN)-(NNN)-(NNNN), “N” refers to non-final position; “N” to final position; and “N” to a non-applicable position for pre-focus. Similarly, in the post-focus positions, since post-low bouncing also seems position-dependent, we divided the string position into two parts: initial vs. non-initial positions. In this string (NNN)-(NNN)-(NNNN), “N” refers to a non-applicable position for post-focus; “N” to non-initial position; and “N” to initial position.

For our statistical analysis, we constructed a linear mixed model implementing the *lmerTest* package (Kuznetsova et al., 2013) in R (R Core Team, 2015). In the focus position, duration, mean intensity, and target pitch were regressed against a model for each tone, where focus was used as a fixed effect, and speaker (5 speakers) and digit (different digits for each tone, except tone 2) were used as random effects^3^. In pre- and post-focus positions, duration, mean intensity, and mean pitch were regressed against a model with string position as a fixed effect and speaker as a random effect. The *Anova* function of the *lmerTest* package was used to test whether a fixed effect was significant, and the *mcp* function from the *lmerTest* package was used for multiple comparison (Tukey) between groups. Here we present the results in the order of focus, pre-focus, and post-focus position. Please note that, for the pre-focus and post-focus positions, we will only provide the results of tone 3 focus since it produced a relatively poorer identification rate than that of the other focused tones.

**Figure 4:**
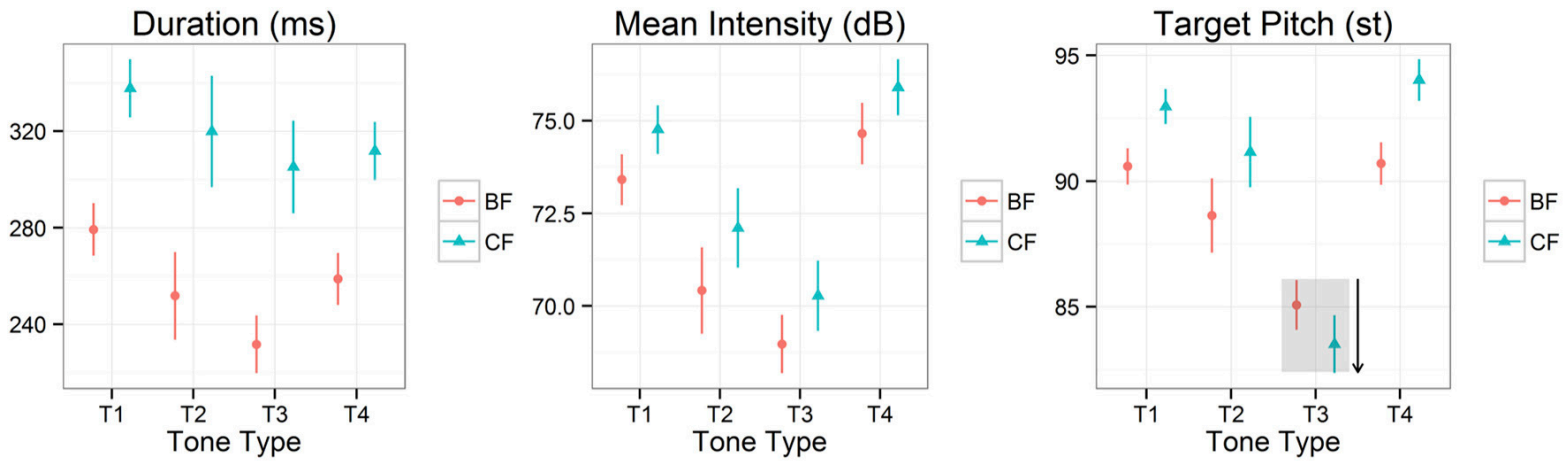
**Duration, mean intensity, and target pitch of all tone types in two focus conditions (BF: broad focus, CF: corrective focus; T1, Tone 1; T2, Tone 2; T3, Tone 3; T4, Tone 4)**. Points indicate mean values and error bars 95% confidence intervals. The downward arrow of the third panel indicates that tone 3 focus is expressed by lowering its pitch point.

Figure 4 plots duration, mean intensity, and target pitch for tones 1–4 in two focus conditions. One interpretation seems simple and clear regarding the prosodic marking of focus—corrective focus exhibits longer duration, higher intensity, and greater pitch range expansion than its broad-focus counterpart for all tone types. Statistical analyses confirmed this visual impression. There existed a significant effect of focus on duration for all tone types, such that corrective focus induced a longer duration than broad focus (T1: *X*^2^ = 64.10, *df* = 1, *p* < 0.001, *R*^2^ = 0.367; T2: *X*^2^ = 22.63, *df* = 1, *p* < 0.001, *R*^2^ = 0.319; T3: *X*^2^ = 47.92, *df* = 1, *p* < 0.001, *R*^2^ = 0.375; T4: *X*^2^ = 48.69, *df* = 1, *p* < 0.001, *R*^2^ = 0.319). Furthermore, the effect of focus on mean intensity was also significant for all tone types, indicating that corrective-focus conditions displayed a higher intensity than their broad-focus counterparts (T1: *X*^2^ = 27.50, *df* = 1, *p* < 0.001, *R*^2^ = 0.733; T2: *X*^2^ = 8.56, *df* = 1, *p* < 0.01, *R*^2^ = 0.527; T3: *X*^2^ = 10.83, *df* = 1, *p* < 0.01, *R*^2^ = 0.601; T4: *X*^2^ = 19.10, *df* = 1, *p* < 0.001, *R*^2^ = 0.764). Finally, focus produced a significant effect on target pitch for all tone types, resulting in corrective focus having greater pitch range expansion than broad focus (T1: *X*^2^ = 43.92, *df* = 1, *p* < 0.001, *R*^2^ = 0.196; T2: *X*^2^ = 34.07, *df* = 1, *p* < 0.001, *R*^2^ = 0.385; T3: *X*^2^ = 14.06, *df* = 1, *p* < 0.001, *R*^2^ = 0.116; T4: *X*^2^ = 83.14, *df* = 1, *p* < 0.001, *R*^2^ = 0.287).

Although the corrective-focus condition showed greater pitch range expansion than the broad-focus for all tone types, we must be sure to consider the smaller size of the pitch range expansion for tone 3 focus. As Table 2 illustrates, the size of the pitch range expansion was only 1.56 st for tone 3 focus, whereas it was at least 2.37 st for other focused tones. In order to test whether this numerical difference is statistically valid, we conducted a linear mixed model using the *lmerTest* package (Kuznetsova et al., 2013) in R (R Core Team, 2015). In this model, tone type (i.e., tone 1, tone 2, tone 3, and tone 4) was used as a fixed effect, speaker and digit were included as random effects, and target pitch was used as a dependent variable. We found that tone type had a significant effect on target pitch (*X*^2^ = 14.88, *df* = 3, *p* < 0.01, *R*^2^ = 0.062). Given this finding, a *post-hoc* multiple comparison procedure was conducted to determine which tone type was significantly different; the output of the multiple comparison analysis is shown in Table 3. Examining the results given in Table 3, we can say that tone 3 digits indeed produced a relatively smaller pitch range expansion in marking prosodic focus, although the difference between tone 2 and tone 3 foci is marginally significant. However, this type of difference was not reflected in the other parameters, such as duration (*X*^2^ = 2.10, *df* = 3, *p* = 0.552, *R*^2^ = 0.378) and intensity (*X*^2^ = 0.39, *df* = 3, *p* = 0.941, *R*^2^ = 0.0679), meaning that all tone types showed quite similar increases in marking prosodic focus, except pitch range expansion. Therefore, we speculate that the smaller pitch range expansion for tone 3 focus was due to its unique prosodic structure: tone 3 focus was expressed by lowering the pitch target, which distinguishes it from other focused tones we considered.

**Table 2:** **The duration, mean intensity, and target pitch values of the broad-focus and corrective-focus condition for each tone type (BF and CF are abbreviations for broad focus and corrective focus, respectively; B-A means the difference between corrective focus and broad focus)**.

	**BF (A)**	**CF (B)**	**B–A**
**DURATION (ms)**			
Tone 1	279.27	337.75	58.48
Tone 2	251.77	319.83	68.06
Tone 3	231.65	305.14	73.49
Tone 4	258.76	311.76	53.00
**MEAN INTENSITY (dB)**			
Tone 1	73.41	74.76	1.35
Tone 2	70.42	72.11	1.69
Tone 3	68.97	70.27	1.30
Tone 4	74.65	75.90	1.25
**TARGET PITCH (st)**			
Tone 1	90.59	92.96	2.37
Tone 2	88.63	91.15	2.52
Tone 3	85.07	83.51	−1.56
Tone 4	90.70	94.02	3.32

**Table 3:** **The output of multiple comparison analysis for target pitch between tone types (Estimate, coefficient estimates; S.E., standard errors)**.

	**Estimate**	**S.E**.	***z*-value**	***p*-value**	
Tone 2 vs. Tone 1	0.1498	0.3824	0.392	0.97902	
Tone 3 vs. Tone 1	−0.8099	0.2962	−2.734	0.03071	^*^
Tone 4 vs. Tone 1	0.9498	0.2613	3.636	0.00164	^**^
Tone 3 vs. Tone 2	−0.9597	0.4190	−2.291	0.09703	.
Tone 4 vs. Tone 2	0.8000	0.3950	2.025	0.17353	
Tone 4 vs. Tone 3	1.7597	0.3123	5.635	< 0.001	^***^

. 0.1 < p < 0.05;

* p < 0.05;

** p < 0.01;

*** p < 0.001.

**Figure 5:**
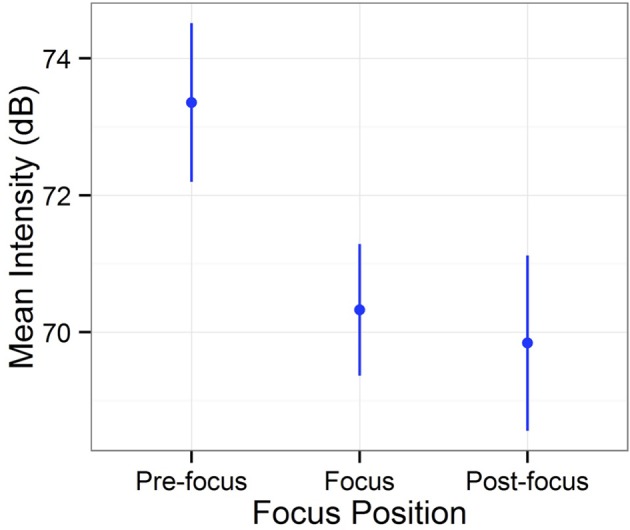
**Mean intensity of the three focus positions: pre-focus, focus, and post-focus**. Points indicate mean values and error bars 95% confidence intervals.

Furthermore, it is worth noting that a tone 3 syllable produced the lowest intensity values in both broad-focus and corrective-focus conditions. The lowest intensity is deemed another unique characteristic of a tone 3 syllable in Mandarin. In order to test if this was so, we aggregated intensity values for each of the three focus positions: pre-focus, focus, and post-focus^4^ (Figure 5), and conducted a linear mixed model using the *lmerTest* (Kuznetsova et al., 2013) in R (R Core Team, 2015) with position as a fixed effect, speaker and digit as random effects, and intensity as a dependent variable. Through this procedure, we found that position had a significant effect on intensity (*X*^2^ = 18.65, *df* = 2, *p* < 0.001, *R*^2^ = 0.729). In order to further explore this result and to identify which position produced a significantly lower intensity, we conducted a *post-hoc* multiple comparison test. The results, as presented in Table 4, revealed that a pre-focus position produced a significantly greater intensity than both focus and post-focus positions, whereas there was no significant difference in intensity between focus and post-focus positions. The results clearly indicate that tone 3 focus did not produce a greater intensity value in marking prosodic focus when compared to adjacent positions.

**Table 4:** **The output of multiple comparison analysis for intensity of tone 3 focus between the three focus positions (Estimate, coefficient estimates; S.E., standard errors)**.

	**Estimate**	**S.E**.	***z*-value**	***p*-value**	
Pre-focus vs. focus	3.1899	0.8749	3.646	< 0.001	^***^
Post-focus vs. focus	1.3723	1.1104	1.236	0.4173	
Pre-focus vs. post-focus	1.8177	0.7015	2.591	0.0238	^*^

* p < 0.05;

*** p < 0.001.

**Figure 6:**
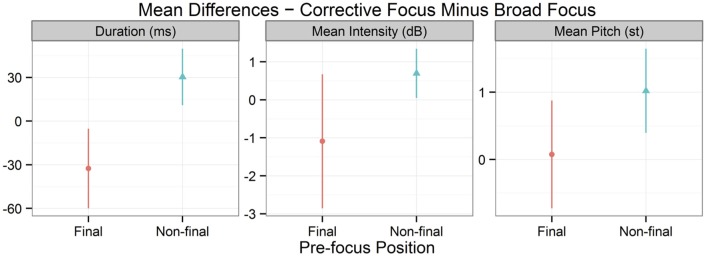
**Duration, mean intensity, and mean pitch values of the pre-focus position of tone 3 digits, separated by final vs. non-final positions**. BF and CF are abbreviations for broad focus and corrective focus, respectively. Points indicate mean values and error bars 95% confidence intervals.

Moving on to consider the pre-focus position of tone 3 digits, Figure 6 plots a simple comparison of the differences in duration, mean intensity, and mean pitch of the two focus conditions, which are separated by final vs. non-final positions. In this figure, the average of each point was determined by subtracting the paired values between broad focus and corrective focus. As shown in Figure 6, the non-final positions had longer duration, higher intensity, and a higher mean pitch than the final positions. In other words, focused tone 3 digits increased the duration, mean intensity, and mean pitch values of their pre-focus position in non-final position. This indicates that the pre-low raising effect was contingent on string position; it only occurred within the same phrase. The results from the linear mixed models partly supported this observation. There were significant effects of string position on both duration (*X*^2^ = 16.62, *df* = 1, *p* < 0.001, *R*^2^ = 0.604) and mean intensity (*X*^2^ = 5.96, *df* = 1, *p* < 0.05, *R*^2^ = 0.124). Although non-final positions had greater mean pitch values than final positions, the effect of string position on mean pitch showed a positive trend but failed to achieve a customary level of statistical significance (*X*^2^ = 2.80, *df* = 1, *p* = 0.09, *R*^2^ = 0.253)^5^.

**Figure 7:**
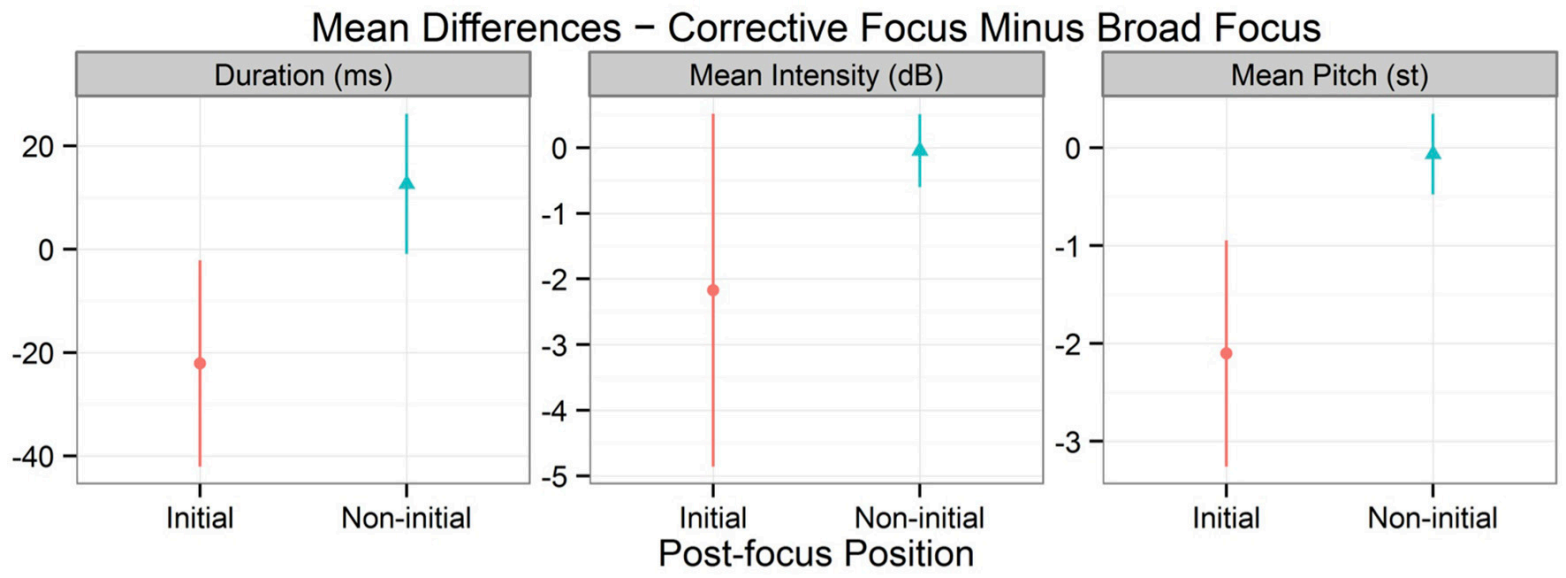
**Duration, mean intensity, and mean pitch values of the post-focus position of tone 3 digits, separated by initial vs. non-initial positions**. BF and CF are abbreviations for broad focus and corrective focus, respectively. Points indicate mean values and error bars 95% confidence intervals.

As for the post-focus position of tone 3 digits, Figure 7 describes the differences between initial vs. non-initial positions by aggregating the following parameters: duration, mean intensity, and mean pitch. Each point of Figure 7 refers to the average value calculated by subtracting the values between broad focus and corrective focus. It is likely that the different post-focus positions reveal different kinds of post-focus effects. In initial positions, corrective-focus conditions clearly showed post-focus compression with reduced duration, mean intensity, and mean pitch values. However, non-initial positions did not show this post-focus compression. Rather, the duration, mean intensity, and mean pitch values showed a rebound effect immediately after tone 3 focus; therefore, the values of these parameters were close to zero, meaning that the differences between the broad-focus and corrective-focus conditions were minimal in the non-initial post-focus conditions. Statistical analyses confirmed this observation for all the parameters (duration: *X*^2^ = 11.25, *df* = 1, *p* < 0.001, *R*^2^ = 0.583; mean intensity: *X*^2^ = 6.77, *df* = 1, *p* < 0.01, *R*^2^ = 0.140; mean pitch: *X*^2^ = 20.22, *df* = 1, *p* < 0.001, *R*^2^ = 0.521). The results clearly suggest that tone 3 focus had different post-focus effects depending on the position in a digit string: the initial post-focus positions showed the post-focus compression effect, whereas the non-initial post-focus positions had the post-low bouncing effect.

**Figure 8:**
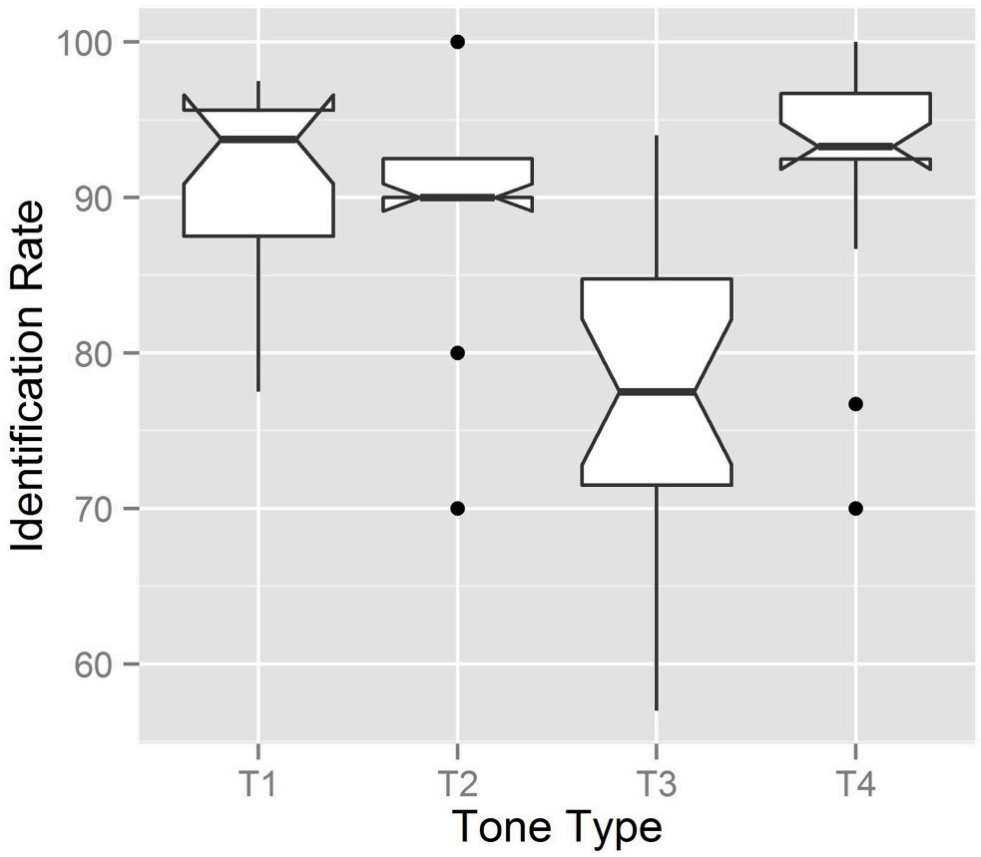
**Boxplot of the identification rate for each tone type**.

## Perception

From the production experiment, we found that tone 3 focus was clearly marked by increased duration, intensity, and pitch range expansion. At the same time, we found that pre-low raising and post-low bouncing effects appeared only within the same phrase. Therefore, this perception experiment was aimed at examining whether listeners could successfully identify tone 3 focus or whether the local dissimilatory effects hindered the recognition of tone 3 focus.

### Data collection

From the production data, we used 100 10-digit strings produced with tone 3 focus [2 tone 3 digits (5, 9) × 10 string positions × 5 speakers], and also randomly selected 80 strings with other focused tones used for both distractors and reference data. The 80 strings were selected so that each tone had an equal number of focus tokens for each string position. A total of 180 strings were randomized and divided into two blocks of 90 strings each, and there was a short break between the two blocks.

Twenty native Mandarin listeners were seated in front of a computer monitor and tested in a quiet room at Tongji University. The audio stimuli were presented to listeners through Sennheiser PC166 headset speakers using Paradigm software (Perception Research Systems, 2007). Before the actual test, we presented three practice trials to listeners to familiarize them with the procedure. In the actual sessions, participants first heard only the phrase with a correction, and were asked to choose which digit was corrected by using a computer mouse. The total length of the perception experiment was around 40 min.

### Results

Table 5 exhibits the identification rate of corrected digits; columns correspond to positions within a string, and rows represent tones 1–4. The overall identification rate was 90.8% for tone 1, 90.5% for tone 2, 77.1% for tone 3, and 92.5% for tone 4^6^. In order to test whether the numerical difference between identification rates was indeed statistically supported, we conducted a logistic regression analysis with tone type as a fixed effect, speaker and digit as random effects, and identification as a dependent variable. From the results, we found that the identification rate actually differed by tone type (*X*^2^ = 134.48, *df* = 3, *p* < 0.001, *R*^2^ = 0.126). We thus conducted a *post-hoc* multiple comparison analysis to determine which tone type received a significantly lower identification rate: Table 7 presents the output of a multiple comparison analysis between tone types. As Table 7 indicates, tone 3 focus, compared with other focused tones, was identified at a significantly lower rate^7^. Some plausible explanations for this low identification rate are detailed in the confusion matrix (Table 8), which contains more in-depth information about the classifier performance of tone 3 focus. Confusion matrices of other focused tones are also presented as reference data.

**Table 5:** **Position-by-position identification rates of tones 1–4**.

** 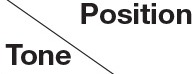 **	**1**	**2**	**3**	**4**	**5**	**6**	**7**	**8**	**9**	**10**	**Average**
Tone 1	95.0	82.5	88.8	97.5	98.8	88.8	98.8	95.0	98.8	63.8	90.8
Tone 2	85.0	95.0	90.0	90.0	75.0	100.0	80.0	100.0	95.0	95.0	90.5
Tone 3	70.5	76.5	75.5	76.0	78.0	79.0	88.0	75.5	88.5	63.0	77.1
Tone 4	91.7	91.7	96.7	91.7	96.7	95.0	96.7	91.7	98.3	75.0	92.5

Numbers represent identification rates (percentage values).

**Table 6:** **The maximum, minimum, and mean identification rate of each tone type (percentage values)**.

**Tone type**	**Max**	**Min**	**Mean**	**SD**
T1	97.5	77.5	90.8	6.8
T2	100.0	70.0	90.5	7.6
T4	100.0	70.0	92.5	7.5
T3	94.0	57.0	77.1	10.8

SD refers to standard deviation.

**Table 7:** **The output of multiple comparison analysis for identification between tone types (Estimate, coefficient estimates; S.E., standard errors)**.

	**Estimate**	**S.E**.	***z*-value**	***p*-value**	
Tone 2 vs. Tone 1	−0.03131	0.27873	−0.112	0.999	
Tone 3 vs. Tone 1	−1.14216	0.14160	−8.066	< 0.001	^***^
Tone 4 vs. Tone 1	0.24220	0.20295	1.193	0.614	
Tone 3 vs. Tone 2	−1.11085	0.25707	−4.321	< 0.001	^***^
Tone 4 vs. Tone 2	0.27352	0.29535	0.926	0.779	
Tone 4 vs. Tone 3	1.38436	0.17209	8.045	< 0.001	^***^

*** p < 0.001.

**Table 8:** **Confusion matrix of corrective focus perception for each focused tone (Top left panel: tone 1, top right panel: tone 2, bottom left panel: tone 3, bottom right panel: tone 4)**.

**Target**	**Perceived**																			
	**1**	**2**	**3**	**4**	**5**	**6**	**7**	**8**	**9**	**10**	**1**	**2**	**3**	**4**	**5**	**6**	**7**	**8**	**9**	**10**
1	95.0	3.8	0.0	0.0	0.0	0.0	1.3	0.0	0.0	0.0	85.0	15.0	0.0	0.0	0.0	0.0	0.0	0.0	0.0	0.0
2	1.3	82.5	6.3	1.3	3.8	0.0	3.8	0.0	1.3	0.0	0.0	95.0	5.0	0.0	0.0	0.0	0.0	0.0	0.0	0.0
3	3.8	3.8	88.8	2.5	0.0	0.0	1.3	0.0	0.0	0.0	0.0	0.0	90.0	10.0	0.0	0.0	0.0	0.0	0.0	0.0
4	0.0	1.3	1.3	97.5	0.0	0.0	0.0	0.0	0.0	0.0	0.0	0.0	0.0	90.0	10.0	0.0	0.0	0.0	0.0	0.0
5	0.0	0.0	0.0	0.0	98.8	1.3	0.0	0.0	0.0	0.0	0.0	0.0	5.0	5.0	75.0	15.0	0.0	0.0	0.0	0.0
6	3.8	0.0	1.3	1.3	2.5	88.8	1.3	1.3	0.0	0.0	0.0	0.0	0.0	0.0	0.0	100.0	0.0	0.0	0.0	0.0
7	0.0	0.0	0.0	0.0	0.0	0.0	98.8	0.0	1.3	0.0	5.0	0.0	0.0	5.0	0.0	5.0	80.0	5.0	0.0	0.0
8	0.0	0.0	1.3	0.0	0.0	0.0	2.5	95.0	1.3	0.0	0.0	0.0	0.0	0.0	0.0	0.0	0.0	100.0	0.0	0.0
9	1.3	0.0	0.0	0.0	0.0	0.0	0.0	0.0	98.8	0.0	0.0	0.0	0.0	0.0	0.0	0.0	0.0	0.0	95.0	5.0
10	2.5	6.3	3.8	1.3	0.0	0.0	2.5	2.5	17.5	63.8	0.0	0.0	0.0	0.0	0.0	5.0	0.0	0.0	0.0	95.0
1	70.5	5.5	6.0	6.0	1.5	3.0	5.0	0.0	1.5	1.0	91.7	6.7	1.7	0.0	0.0	0.0	0.0	0.0	0.0	0.0
2	6.0	76.5	9.5	4.0	1.0	0.5	0.0	1.5	0.0	1.0	5.0	91.7	0.0	0.0	0.0	0.0	1.7	1.7	0.0	0.0
3	3.5	15.0	75.5	0.5	1.5	2.0	2.0	0.0	0.0	0.0	1.7	1.7	96.7	0.0	0.0	0.0	0.0	0.0	0.0	0.0
4	1.5	0.5	0.0	76.0	9.0	8.0	4.0	0.0	0.0	1.0	1.7	0.0	1.7	91.7	3.3	1.7	0.0	0.0	0.0	0.0
5	0.0	0.0	1.0	17.5	78.0	1.5	0.5	0.5	1.0	0.0	1.7	0.0	0.0	1.7	96.7	0.0	0.0	0.0	0.0	0.0
6	1.0	2.0	2.0	1.5	11.0	79.0	3.0	0.0	0.0	0.5	0.0	0.0	0.0	0.0	3.3	95.0	1.7	0.0	0.0	0.0
7	0.0	0.0	2.0	1.5	3.0	0.5	88.0	4.5	0.5	0.0	0.0	1.7	1.7	0.0	0.0	0.0	96.7	0.0	0.0	0.0
8	0.5	1.0	0.0	0.5	0.5	1.0	19.5	75.5	1.0	0.5	0.0	0.0	0.0	0.0	0.0	0.0	8.3	91.7	0.0	0.0
9	1.0	0.0	0.5	0.5	0.0	0.0	2.0	7.5	88.5	0.0	0.0	0.0	0.0	0.0	0.0	0.0	1.7	0.0	98.3	0.0
10	1.0	4.0	1.5	2.0	0.0	1.0	4.5	1.0	22.0	63.0	0.0	0.0	0.0	0.0	5.0	0.0	10.0	3.3	6.7	75.0

Cells highlighted in black indicate focused positions. Cells highlighted in gray refer to pre- or post-focus positions across the phrase boundary for tone 3 focus. Vertical lines refer to phrase boundaries. Numbers represent identification rates (percentage values).

As Table 8 below shows, there exists a systematic difference between tone 3 focus and other focused tones. Incorrect answers usually occurred within the same phrase, immediately preceding and/or following the focus position for tone 3 focus, but those incorrect answers were not often present for other focused tones^8^. The results suggest that local dissimilation is a unique phenomenon found in the pre- and post-focus positions within the same phrase when a tone 3 syllable is focused.

Let us now refer to tone 3 focus in Table 8. First, we will examine the rate of incorrect answers in the pre-focus positions, followed by an explanation of the post-focus positions. When position 3 was focused, listeners chose position 2 15.0% of the time. When position 5 was focused, listeners chose position 4 17.5% of the time. When position 6 was focused, position 5 was chosen 11.0% of the time. When position 8 was focused, listeners chose position 7 at a rate of 19.5%. When position 10 was focused, listeners chose position 9 at a rate of 22.0%. Overall, the average rate of incorrect answers was 14.1% for the pre-focus position within the same phrase. Post-focus positions also showed a degree of confusion; however, this was to a lesser extent. The average rate of incorrect performance was 5.2% for a post-focus position within the same phrase. Even if we score by phrase rather than position, the identification rate of tone 3 focus would increase from 77.1 to 96.4%, suggesting that the confusion of tone 3 focus was indeed due to pre-focus raising and/or post-low bouncing effects within the same phrase. On the contrary, the confusion rate of tone 3 focus was very little or minimal across the phrase boundary. When position 3 was focused, listeners chose position 4 only 0.5% of the time. When position 6 was focused, listeners chose position 7 only 3.0% of the time. When position 4 was focused, listeners did not choose position 3 at all. When position 7 was focused, listeners chose position 6 only 0.5% of the time. Therefore, we can say that the pre-focus raising and/or post-low bouncing effects hindered the recognition of tone 3 focus within the same phrase but not across the phrase boundary, meaning that the lower identification rate of tone 3 focus was due to the interaction between local dissimilatory and phrase boundary effects.

## Discussion

The aims of this study were twofold: (a) to investigate the prosodic nature of tone 3 focus and its within-phrase local dissimilatory effects; and (b) to examine whether listeners successfully identify tone 3 focus, or whether the local dissimilatory effects present within the same phrase hinder the recognition of tone 3 focus. The method developed in this study allowed a systematic investigation of tone 3 focus and its within-phrase local dissimilatory effects. We have observed that production and perception results for tone 3 focus were compatible with each other.

In production, tone 3 focus was realized with increased duration, intensity, and pitch range expansion similar to other focused tones. Some interesting phenomena with tone 3 focus included local dissimilatory effects—pre-low raising and post-low bouncing effects were present within the same phrase but absent across the phrase boundary. In perception, tone 3 focus received relatively low identification rates due to the interaction between local dissimilatory and phrase boundary effects when compared to other focused tones—incorrect answers most likely occurred in the immediate pre- or post-focus position within the same phrase.

In this study, the key issue at hand was to ascertain why tone 3 focus achieved low identification rates. From the perception data, we observed that the local dissimilatory effects of tone 3 focus (i.e., pre-low raising and post-low bouncing) clearly hindered the identification of tone 3 focus within the same phrase. In addition to these, there are at least two more reasons for the low identification rate: (a) smaller capacity for pitch range expansion; and (b) tone 3's low intensity by nature. We discuss these in turn below.

First, lowering the pitch target results in a smaller capacity for pitch range expansion. Although other focused tones were expressed by pitch raising, tone 3 focus was expressed by pitch lowering. We assume that from a physiological point of view, there is more limitation on lowering the low pitch of tone 3 than raising the high pitch of other tones given that human pitch range is within 100 Hz (Baken and Orlikoff, 2000; Keating and Kuo, 2012; Kuang, 2013), and a tone 3 syllable is produced at the floor of the pitch range (Kuang, p.c). Our production data support this, given that tone 3 focus showed just 1.56 st for pitch range expansion, while other focused tones showed a minimum of 2.37 st (tone 1: 2.37, tone 2: 2.52, tone 4: 3.32). Accordingly, the present study is in favor of Wang et al.'s (2002) finding that raising a high pitch target is a more important perceptual cue for identifying focus and seems to support Shen's (1992) theory that a top line of the pitch contour cues focus.

Another speculation is that tone 3 digits produce low intensity by nature. As previously shown in Table 2, tone 3 digits in the corrective-focus condition produced an average of 70.3 decibels, which is even smaller than the intensity of other tones in the broad-focus condition. Furthermore, as illustrated by Table 4, tone 3 digits did not employ intensity effectively when marking focus; the pre-focus position produced a significantly greater intensity value than the focus position, and the intensity of the post-focus position was significantly equivalent with that of the focus position. In our experiment, focused tone 3 digits were always surrounded by other tones. This positioning is because of the fact that, in Mandarin, multiple tone 3 digits cannot appear consecutively due to tone sandhi—tone 3 becomes tone 2 when followed by another tone 3. Therefore, we posit that the (seemingly) greater or equivalent intensity of other adjacent tones may also, at least to some extent, affect the identification of tone 3 focus.

Although this study revealed interesting results about tone 3 focus when compared with other focused tones, there were several limitations in the way the study was conducted. First, given that a limited set of phone-number strings were used repeatedly in the stimuli, the production task of this study is not entirely devoid of the issue of anaphoric de-stressing. Second, since corrected digits were underlined and highlighted in boldface type in the production task, there is a possibility that the generalizability to naturalistic uses of corrective focus could be quite limited. Finally, as already stated in footnote 2, the small sample number of speakers is a clear limitation of this study. In future research, more speakers need to be recruited in order to increase the generalizability of the study and improve the statistical power of production data.

In summary, this study enabled us to untangle the prosodic nature of tone 3 focus and its within-phrase local dissimilatory effects. We found that although focused tone 3 digits were clearly marked by greater duration, intensity, and pitch range expansion, the identification rate of tone 3 focus was not as high as other focused tones due to the interaction between local dissimilatory and phrase boundary effects, a smaller capacity for pitch range expansion, and weak intensity by nature. The results of our study indicate that although tone 3 did in fact show clearly present acoustic correlates of focus, an important issue is that the identification of tone 3 focus was not universally recoverable by listeners despite being explicitly employed by speakers. Previous studies have claimed that (purely) prosodic marking of focus is clear in Mandarin (e.g., Yuan, 2004; Chen et al., 2009; Liu, 2009). However, we found that even within a language where prosodic marking of focus is clear, the location of prosodic focus can be difficult to identify under certain circumstances depending on the tone type. Therefore, we maintain that purely prosodic marking of focus can differ, even within a given language. Instead of remaining uniform, it behaves differently, conforming to the prosodic system of each language. Further examination of this occurrence will focus on languages with tonal patterns similar to tone 3 in Mandarin (e.g., Cantonese, Hakka) and languages where focus is characterized by a low pitch target (e.g., Turkish).

## Author contributions

YL conducted a production experiment, analyzed the data and wrote the paper. TW conducted a perception experiment and collected the perception data. ML designed the experiment designs for both production and perception experiments and overviewed the paper.

### Conflict of interest statement

The authors declare that the research was conducted in the absence of any commercial or financial relationships that could be construed as a potential conflict of interest.

## Footnotes

^1^Broad focus refers to a condition where no single element receives focus in a sentence.

^2^In this study, we assume that pre and post dashes represent the phrase boundaries in the phone-number string expressed in (NNN)-(NNN)-(NNNN). Our assumption is based on the observation that speakers had a strong tendency to place a phrase boundary before the dashes in the phone-number string. Related to this, the expression “the same phrase” refers to the phrase within parentheses in (NNN)-(NNN)-(NNNN). Please note that throughout the presentation of the results, we use the notions of “phrase boundaries” and “the same phrase” based on the objective terms in the phone-number string.

^3^Although we treated speaker as a random effect, one may wonder about possible speaker effects when marking prosodic focus given that the sample number of speakers was small in this study. The results of the *Anova* function of the *lmerTest* package showed that there were no speaker effects for the parameters of intensity (*X*^2^ = 0.81, *df* = 1, *p* = 0.368) and pitch range (*X*^2^ = 0.002, *df* = 1, *p* = 0.961), but there was a speaker effect for duration (*X*^2^ = 181.03, *df* = 1, *p* < 0.001). We further investigated whether the significant effect of duration was smaller, stronger, or absent for certain speakers. Figure 3 shows the speaker-by-speaker difference calculated by corrective focus minus broad focus. As Figure 3 illustrates, the speakers employed duration differently when marking focus. For example, Speaker 1's duration effect was the smallest, Speaker 5's duration effect was the strongest, and the other speakers' duration effects fell somewhere in between. Although the duration effect varied among speakers, the results of a t-test indicated that the difference by each speaker turned out to be highly significant. This suggests that corrective focus was successfully discriminated from broad focus by all the speakers by means of duration.

^4^We measured the intensity of pre- and post-focus positions only within the same phrase. In other words, in the 10-digit string (NNN)-(NNN)-(NNNN), the pre-focus position – labeled “N” – was excluded since its positioning appears across the phrase boundary and the final position – labeled “N” – was also excluded since this position is not applicable for pre-focus. Measurements of intensity for the post-focus position were conducted in the same way.

^5^One of the reviewers pointed out that if this study had included more subjects, the statistical power would have been greater. We noted that the small number of speakers is a limitation in our study.

^6^One may wonder whether the identification rate of tone 3 focus was stabilized among listeners. As Figure 8 and Table 6 present, among the four tone types, tone 3 focus had the lowest identification rate and the greatest standard deviation. The high standard deviation of tone 3 focus indicates a greatly varying identification rate among listeners. For example, some listeners (listener 9 and listener 11) showed identification rates of 68 and 57%, respectively, whereas others (listener 10 and listener 11) showed identification rates of 94 and 92%, respectively. These results imply that some listeners had difficulty identifying tone 3 focus when compared to other focused tones, but others did not show such difficulty.

^7^As previously mentioned, we included 100 10-digit strings produced in production, which includes all the corrected digits from tones 1–4. We also added 80 more phone-number strings produced for tone 3 focus to the list. Therefore, we ended up with a total of 180 phone-number strings, resulting in a ratio of 5:4 of targets to distractors. Although the high proportion of tone 3 focus items may potentially introduce a bias toward tone 3 responses, the specific direction of the observed effects suggest that such an effect cannot explain our results.

^8^One may argue that incorrect answers also appeared within the same phrase especially when positions 1 and 5 were focused. Although this observation is correct, the pattern should not be conclusive given that it is neither consistent nor straightforward across all the focused positions of tone 2 focus.

## References

[B1] Baken R. J. Orlikoff R. F. (2000). Clinical Measurement of Speech and Voice. San Diego, CA: Singular Publishing Group.

[B2] Cao J. (2002). 汉语声调与语调的关系 [The relationship between tone and intonation in Mandarin Chinese]. Zhongguo Yuwen 3, 195–202.

[B3] Cao J. (2012). Pitch prominence and tonal typology for low register tone in Mandarin, in Proceedings of the 3rd International Symposium on Tonal Aspects of Languages (Nanjing).

[B4] Cao W. Zhang J. (2008). Tone-3 accent realization in short Chinese sentences. Tsinghua Sci. Technol. 13, 533–539. 10.1016/S1007-0214(08)70085-3

[B5] Chao Y. R. (1968). A Grammar of Spoken Chinese. Berkeley, CA: University of California Press.

[B6] Chen A. (2012). Prosodic investigation on information structure, in The Expression of Information Structure, eds Krifka M. Musan R. (Berlin: De Gruyter Mouton), 251–286.

[B7] Chen S. Wang B. Xu Y. (2009). Closely related languages, different ways of realizing focus, in Proceedings of Interspeech (Brighton), 1007–1010.

[B8] Chen Y. Gussenhoven C. (2008). Emphasis and tonal implementation in Standard Chinese. J. Phon. 36, 724–746. 10.1016/j.wocn.2008.06.003

[B9] Cohan J. B. (2000). The Realization and Function of Focus in Spoken English. Ph.D. thesis, The University of Texas, Austin.

[B10] Greif M. (2010). Contrastive focus in Mandarin Chinese, in Proceedings of Speech Prosody (Chicago, IL), 2–5.

[B11] Halliday M. A. K. (1967). Intonation and Grammar in British English. The Hague, NL: Mouton.

[B12] Kabagema-Bilan E. López-Jiménez B. Truckenbrodt H. (2011). Multiple focus in Mandarin Chinese. Lingua 121, 1890–1905. 10.1016/j.lingua.2011.02.005

[B13] Keating P. Kuo G. (2012). Comparison of speaking fundamental frequency in English and Mandarin. J. Acoust. Soc. Am. 132, 1050–1060. 10.1121/1.4730893 22894225

[B14] Kuang J. (2013). Phonation in Tonal Contrasts. Ph.D. thesis, University of California, Los Angeles.

[B15] Kuznetsova A. Brockhoff P. B. Christensen H. B. (2013). lmerTest: Tests for Random and Fixed Effects for Linear Mixed Effect Models (lmer Objects of lme4 package). *R Package Version* Available online at: http://cran.r-project.org/web/packages/lmerTest/index.html

[B16] Ladd D. R. (1996). Intonational Phonology. Cambridge: Cambridge University Press.

[B17] Lee Y.- C. (2015). Prosodic Focus within and across Languages. Ph.D. thesis, University of Pennsylvania.

[B18] Liu F. (2009). Intonation Sytems of Mandarin and English: A Functional Approach. Ph.D. thesis, University of Chicago.

[B19] Liu F. Xu Y. (2005). Parallel encoding of focus and interrogative meaning in Mandarin intonation. Phonetica 62, 70–87. 10.1159/000090090 16391495

[B20] Liu F. Xu Y. (2007). The neutral tone in question intonation in Mandarin, in Proceediings of Interspeech (Antwerp), 630–633.

[B21] Perception Research Systems 2007. Perception Research Systems. (2007). Paradigm Stimulus Presentation. Available online at: http://www.paradigmexperiments.com

[B22] Prom-on S. Liu F. Xu Y. (2012). Post-low bouncing in Mandarin Chinese : acoustic analysis and computational modeling. J. Acoust. Soc. Am. 132, 421–432. 10.1121/1.4725762 22779489

[B23] R Core Team (2015). A Language and Environment for Statistical Computing. Vienna: R Foundation for Statistical Computing.

[B24] Shen J. (1992). Hanyu yudiao moxing chuyi [On Chinese intonation model]. Yuwen Yanjiu 45, 16–24.

[B25] Shih C. (1988). Tone and intonation in Mandarin, in Working Papers of the Cornell Phonetics Laboratory, Vol. 3, (Ithaca, NY), 83–109.

[B26] Wang B. (2002). **汉语韵律知觉的研究** [The Research on Perception of Prosody in Mandarin]. Ph.D. thesis, The Institute of Psychology, Chinese Academy of Sciences.

[B27] Wang B. Lu S. Yang Y. (2002). 汉语语句中重读音节音高变化模式研究 [The pitch movement of stressed syllable in Chinese sentences]. Acta Acustica 27, 234–240.

[B28] Wang B. Xu Y. (2011). Differential prosodic encoding of topic and focus in sentence-initial position in Mandarin Chinese. J. Phon. 39, 595–611. 10.1016/j.wocn.2011.03.006

[B29] Xu Y. (1997). Contextual tonal variations in Mandarin. J. Phon. 25, 61–83. 10.1006/jpho.1996.0034

[B30] Xu Y. (1999). Effects of tone and focus on the formation and alignment of f0 contours. J. Phon. 27, 55–105. 10.1006/jpho.1999.0086

[B31] Xu Y. (2013). ProsodyPro — A Tool for large-scale systematic prosody analysis, in Proceedings of Tools and Resources for the Analysis of Speech Prosody (Aix-en-Provence), 7–10.

[B32] Xu Y. Chen S. Wang B. (2012). Prosodic focus with and without post-focus compression: a typological divide within the same language family? Linguist. Rev. 29, 131–147. 10.1515/tlr-2012-0006

[B33] Xu Y. Wang M. (2009). Organizing syllables into groups – evidence from F0 and duration patterns in Mandarin. J. Phon. 37, 502–520. 10.1016/j.wocn.2009.08.003 23482405 PMC3589580

[B34] Xu Y. Wang Q. (2001). Pitch targets and their realization: evidence from Mandarin Chinese. Speech Commun. 33, 319–337. 10.1016/S0167-6393(00)00063-7

[B35] Xu Y. Xu C. Sun X. (2004). On the temporal domain of focus, in Proceedings of Speech Prosody (Nara), 81–84.

[B36] Yuan J. (2004). Intonation in Mandarin Chinese: Acoustics, Perception, and Computational Modeling. Ph.D. thesis, Cornell University.

